# Distress and eustress: an analysis of the stress experiences of offshore international students

**DOI:** 10.3389/fpsyg.2023.1144767

**Published:** 2023-05-05

**Authors:** Wuwei Gong, Susan A. Geertshuis

**Affiliations:** Business School, University of Auckland, Auckland, New Zealand

**Keywords:** stress, stressors, perception, distress, eustress, stress management, offshore international students, post-graduate students

## Abstract

**Introduction:**

The popularity of online learning provides higher education institutions with opportunities to deliver remote educational programs for international students who remain in their home countries but enroll in overseas universities. Yet the voices of offshore international students (OISs) have been rarely heard. This study focuses on the stress experiences of OISs, aiming to investigate the perception of stressors, specific responses, and stress management strategies pertaining to distress (negative stress) and eustress (positive stress).

**Methods:**

Semi-structured interviews were conducted in two phases with 18 Chinese postgraduate OISs enrolled in a range of institutions and disciplines. Interviews took place online and were analyzed thematically to explore participants’ experiences.

**Results:**

Stress was found to originate from both socially- and task-based factors, closely related to participants’ need to integrate into their on-campus community and gain useful knowledge and skills. Particular sources of stress were associated with distinct perceptions and subsequent responses and management strategies.

**Discussion:**

A summarizing theoretical model is offered to highlight the separate construct of distress and eustress, indicating tentative causal relationships to extend existing stress models to an educational context and provide new insights into OISs. Practical implications are identified and recommendations are provided for policy-makers, teachers, and students.

## Introduction

This paper investigates the stress-related perceptions, responses, and management strategies of postgraduate international students who study offshore usually in their home countries. In the paragraphs below we review previous research into the stresses experienced by postgraduate international students in general and identify a lack of research into the more particular case of postgraduate offshore international students (OISs). We then describe recent theoretical models of stress before providing an account of our research method.

Stress among postgraduate international students in tertiary education has been found to be related to cultural differences, inconsistent educational expectations (Yan and Berliner, 2013; Liao and Wei, 2014), lack of social support (Zhang and Brunton, 2007; Lai et al., 2020), discrimination (Smith and Khawaja, 2011; Wilczewski et al., 2021), and financial insecurity (Charles and Stewart, 1991; O’Byrne et al., 2021). It has been associated with issues such as anxiety, insomnia, poor academic performance, and dropout (Laufer and Gorup, 2019; Lai et al., 2020; Garbóczy et al., 2021). Common distress-coping strategies reported by postgraduate international students include self-help strategies such as goal setting and wishful thinking (Zhang and Brunton, 2007; Garbóczy et al., 2021) and support-seeking strategies such as obtaining information from the government agencies or the university (O’Byrne et al., 2021).

Wide adoption of online learning strengthens the confidence of educational institutions to continue providing online programs for OISs in exceptional circumstances (Arbaugh et al., 2009; Prasetyanto et al., 2022). However, among studies on the stress of international students, few articles differentiate OISs from onshore international students, although their experiences are likely to be differentiated by learning and living environments. Lai et al. (2020) and Wilczewski et al. (2021) found that offshore students experienced a lower level of living stress when compared to onshore students. This might be because offshore students were located in a context closer to families or friends. However, Choo (2021) points out that OISs may experience challenges that have not been identified and researched, such as courses being taught in English while living in a non-English-speaking environment. The effects of these factors on stress have not been explored, and the voices of OISs have been rarely heard.

Not only have the stress-related experiences of OISs been neglected in recent research, but much of the work on international students in general adopts an orientation to stress that is not in full alignment with recent theory. Stress in much of the educational literature is assumed to be a wholly negative response to negative stimuli which results in negative outcomes such as depression or anxiety (e.g., Lai et al., 2020; Garbóczy et al., 2021). This is not the perspective taken in the wider literature on stress which identifies both positive (eustress) and negative (distress) forms of stress that are associated with differentiated patterns of response (Selye, 1956; Lazarus and Folkman, 1984; Le Fevre et al., 2003; Gibbons et al., 2008). That is, recent research suggests that eustress and distress are perceived, experienced, and managed differently (Lepine et al., 2004; Simmons and Nelson, 2007).

This research sought to explore the eustress and distress-related experiences of OISs in order to provide a rich account of their stress-related experiences. In the following section we describe the conceptual framework that guided our study design.

## Conceptual framework

To facilitate the design of our study and interpretation of our data we brought together two models: the Holistic Stress Model (HSM) (Simmons and Nelson, 2007) and the Challenge-Hindrance Framework (CHF) (Lepine et al., 2004). The HSM seeks to explain stress through the relationship between individual traits, responses, and stress management while the CHF focuses on the associations between external stressors and stress responses.

According to the HSM, stressors are inherently neutral, and it is individual differences that affect the relationship between stressors and responses (Hargrove et al., 2013). The model suggests that when individuals encounter stressors, they may experience eustress or distress, indicated by emotional, attitudinal, or behavioral responses (Simmons and Nelson, 2007). The CHF, however, purports that not all external stressors are neutral. They argue that there can be challenge (positive) or hindrance (negative) stressors which influence individual responses (Lepine et al., 2004; Podsakoff et al., 2007). Challenge stressors contribute to motivation and high performance (LePine et al., 2004). Hindrance stressors limit satisfaction and individual development (Podsakoff et al., 2007).

Despite different focuses on the influential factors of eustress and distress, the two streams of research both recognize perception as the underlying mechanism that leads to stress responses. Based on the emphasis on individual perception, the conceptualization of eustress can be extended to include psychological and physical responses to stressors, which are determined by positive individual perceptions; distress is the psychological and physical responses to stressors, which are determined by negative individual perceptions (Le Fevre et al., 2003). Both models propose causal relationships in that eustress or distress follows a positive or negative perception in response to a stressor. Feelings of eustress may be actively savored or managed in the hope of making them last (Hargrove et al., 2013). Similarly, distress responses may lead to coping and efforts to mitigate the distress response (Lai et al., 2020).

Consistent with these models, this research set out four research questions to explore the stress process experienced by postgraduate OISs:

RQ1. What stressors are identified as triggering different perceptions leading to distress and eustress?
RQ2. What stress responses are reported?
RQ3. How is the management of distress and eustress reported?
RQ4. Are there associations between perceptions of stressors (RQ1), stress responses (RQ2), and stress management strategies (RQ3)?

## Method

### Research design

This research was based on the theoretical perspective of interpretivism (Crotty, 1998). We adopted a phenomenological approach to better understand the lived experiences of study participants through an analysis of their recollections and reflections (Lazarus and Folkman, 1984). The study was conducted in two phases: phase 1 provided for the initial development of a model in answer to the research questions; phase 2 was a replication of phase 1 but with a broader sample of participants. Phase 2 sought to examine the transferability of the answers to the research questions developed during phase 1 (Tuval-Mashiach, 2021).

### Study participants and contexts

In phase 1 of the study, 10 postgraduate OISs from the authors’ university in New Zealand (NZ) were interviewed. In phase 2, a further eight students from other institutions were interviewed. Demographic information is shown in Table 1. All the participants are Chinese, mostly studying from their home country (*n* = 16). Participants had courses conducted either in a hybrid format (i.e., offshore students watched streamed classes delivered on campus to onshore students) (Choo, 2021) or a purely online approach (i.e., both teachers and students work online) (Prasetyanto et al., 2022). For participants having research programs, communication with supervisors was online using tools such as email and Zoom.

**Table 1:** Participant demographic information.

Participant	Major	Host country	Age	Gender
Phase one				
Participant 1	Master of Commerce	NZ	30	Female
Participant 2	Master of Commerce	NZ	26	Female
Participant 3	PhD in Marketing	NZ	32	Female
Participant 4	Exchange Program in Business School	NZ	26	Male
Participant 5	PhD in Marketing	NZ	34	Female
Participant 6	Exchange Program in Business School	NZ	26	Female
Participant 7	Master of Management	NZ	33	Male
Participant 8	Master of Management	NZ	25	Male
Participant 9	Master of Marketing	NZ	24	Female
Participant 10	Master of Accounting	NZ	24	Female
Phase two				
Participant 11	Master of Commerce	Australia	26	Female
Participant 12	Master of Sociology	America	27	Female
Participant 13	Master of Business Analytics	Singapore	22	Male
Participant 14	PhD in Linguistics	Malaysia	48	Female
Participant 15	PhD in Electrical Engineering	Thailand	34	Male
Participant 16	Master of International Relations	Australia	26	Female
Participant 17	Master of Science	British	24	Female
Participant 18	Master of Accounting	Australia	23	Male

### Instrument

To guide semi-structured interviews, an interview schedule was designed, written in both English and Chinese. Each question invited participants to describe scenarios where they felt stressed, and they were encouraged by formal or informal prompts to share details regarding their responses and stress management strategies in their specified contexts (Leech, 2002). Eight interview questions were designed. For example, one question was “Could you think of a time when you felt stressed and positive during study?’’, and one prompt was “Your story is interesting. Could you please tell me more about your reactions in this context?’’. The form of semi-structured, in-depth interview enabled higher flexibility and richer insights yielded through lengthy interviews (Belk et al., 2012; Quinlan et al., 2015).

### Data collection procedure

Upon the approval of the university ethics committee, an audio-recorded semi-structured, in-depth interview was conducted by the first author with each participant via Zoom, WeChat, or telephone. Each interview lasted about 60 min. Chinese, being the native language for both the interviewer and interviewees, was used in interviews to minimize language barriers (Welch and Piekkari, 2006).

Strategies were used to minimize distractions and build rapport with participants during virtual and telephone interviews. To reduce distractions due to noise, the interviewer would close windows and wear earphones during the interview, and interviewees would be politely requested to do the same if they were in a noisy environment (Oliffe et al., 2021). When technical issues arose to influence the quality of communication, the interviewer would slow down her speed of talking and take pause before asking questions to adjust to potential lags (Oliffe et al., 2021). To build rapport with interviewees, the interviewer attempted to display a friendly and active style (Burke and Miller, 2001). With participants’ consent, the interviewer added their social media accounts and had brief interactions prior to the formal interview, introducing the interviewer’s identity and research topic, expressing gratitude, and scheduling the interview (Lo Iacono et al., 2016). Meanwhile, the interviewer examined participants’ posted pictures or videos to gain an impression of their current living and study status. These understandings were used to form effective, personalized icebreaking, rapport-building questions.

Interviews via Zoom were recorded to collect the audio recording for further analysis. All Zoom video recordings were deleted after the interview and only the audio recordings were saved. For interviews via WeChat or phone, the researcher called the participants and recorded their voices on the researcher’s mobile phone. Recordings were saved on a laptop hard drive, a mobile phone, and a cloud service such as WeChat Collection to ensure data storage security.

### Data analysis

Three stages of data preparation and analysis: transcription, translation, and thematic analysis were conducted taking approximately 480 hours over in 3 months. Each of the stages is detailed below.

In the first and second stages, audio data was transcribed into Chinese text and later translated into English by the first author. Firstly, audio recordings were transcribed into Chinese text with an online robot service.^1^ The texts were corrected and then amended based on the principle of de-naturalism. This principle emphasizes the meaning and conception of the interviewee by removing interview noise such as stutters to reduce the bias in interpreting participants’ speech mechanisms (Oliver et al., 2005). Next the Chinese transcript was translated into English. To achieve an accurate and understandable translation, idioms along with the tense of verbs and pronouns (e.g., he, she, it) which were not differentiated in Chinese oral speaking were edited according to the context or explained in subsequent brackets (Choi et al., 2012). At the end of the second stage, the transcripts were cross-checked (Halcomb and Davidson, 2006), by providing the English and Chinese transcripts to participants to check for accuracy. Later, with the consent of participants, follow-up interviews were conducted with all participants on WeChat to clarify ambiguities or expand noteworthy experiences noticed during transcription and translation.

In the third stage, qualitative thematic analysis was applied by both researchers to find patterns of repeated meaning and identify themes in relation to research questions (Braun and Clarke, 2006). After coding all English transcripts in NVivo, codes were classified into three codebooks to facilitate theme identification. That is, initial data codes were parsed to identify perceptions of stressors (RQ1), responses (RQ2), and management strategies (RQ3). When all data were coded and assigned to relevant codebooks, the researchers began sorting codes into relevant clusters and considering emerging themes and sub-themes (Braun and Clarke, 2006). The authors examined transcripts, notes, extracts of data, codes, and themes to specify the essence of each theme (Hycner, 1985).

One hundred and thirty-seven codes were discovered from the first data collection phase with 10 interviews. Among these codes, only six new codes were identified from the last three interviews and none of these new codes suggested a new sub-theme (Guest et al., 2006). This indicates that the last three interviews produced little new information to address the research questions, and it was justifiable to argue that a sample size of 10 with in-depth interviews was adequate for theme identification (Guest et al., 2006). Following the second phase of data collection, data was analyzed based on the previously identified coding system. Data was also re-examined for previously unidentified themes but none were found. In the subsequent findings and discussion sections both data sets are considered together.

### Researcher positionality

In accordance with the robust practice in qualitative research we acknowledge the influence of our own positionality in conducting this research (Twining et al., 2017). The authors are both closely involved in the research topic and therefore their values and beliefs will have affected this research (Holmes, 2020). The first author approached the research problem and interviews having experienced being an OIS. The second author approached the research problem having taught in the hybrid mode to on-campus students and OISs. This configuration enabled the researchers to integrate emic and etic perspectives and engage in a degree of reflexivity which might not have been possible had they worked alone (Morris et al., 1999).

## Findings

### RQ1. What stressors are identified as triggering different perceptions leading to distress and eustress?

Four themes of perceptions were identified and named as follows: Rejecting, Caring, Constraining, and Stretching. Each is described below. We note that two of the four themes are positive, eustress-related (Caring and Stretching) and two are negative, distress-related (Rejecting and Constraining); two relate to social experiences (Rejecting and Caring) and two relate to studying tasks (Constraining and Stretching).

#### Rejecting

Stressors in this context were perceived by participants as indicating they did not belong or were disconnected from the dominant on-campus student group. The stressors originated from two main sources, teaching staff and technologies.

Firstly, while attending hybrid courses, participants perceived themselves to be rejected when they were treated differently and more poorly than their on-campus peers:

The interaction (with students online) was definitely less than with on-campus students… Also, the teacher cared (more) about on-campus students during (in-class) discussions. We (online students) completely did discussions by ourselves. (Participant 17)

Sometimes participants could not interact with the teacher during classes but were only provided with recordings which added to their feelings of rejection:

I had one course bad in particular… Really bad. Every time they (teacher and on-campus students) just had classes by themselves… Then they (the teacher) would (only) send recordings for us to watch…We didn’t have a sense of participation, not at all. And she often didn’t reply to emails; sometimes (her) attitudes were bad (impatient) in particular (when replying). (Participant 2)

Further perceptions of rejection originated with technological problems which left participants feeling alienated and unimportant. Participants’ perception was that if OISs mattered, these technical difficulties would have been resolved:

I could not hear the voice of on-campus people because they were too far away from the computer (used to collect voices). (Participant 11)

Maybe (what the teacher) writes on the blackboard cannot be seen as well. (Participant 3)

Similarly, due to Chinese political restrictions, some teaching programs could only be accessed with a VPN. Compulsory usage of the permitted but unstable university-provided VPN evoked participants’ impression that they were not important and were not given equal opportunities to succeed:

(The exam system) could only be connected by the university VPN. But the university VPN would be disconnected when so many students were taking exams online at the same time… (When logging back) you (students) would find the exam was expired, and you could not get in again. (Participant 10)

#### Caring

A second theme related to perceptions of stressors (RQ1) was that of caring. Caring stressors originated from two aspects, interacting with teaching staff and connecting with other students.

Participants reported positive impressions of teachers who remembered offshore students and accommodated their needs. This was particularly apparent in accounts from participants who contrasted these teachers with others who they perceived as rejecting:

Recently when my supervisor heard my father passed away, (the supervisor) wrote an email as a comfort… According to what I know, my supervisor is relatively positive, giving us (OISs) several online group meetings. Other supervisors may do less in this aspect (holding online meetings). (Participant 14)

Perceptions of caring extended to positive perceptions of networking with fellow students and from extra-curricular university support services:

You (I) could talk with him (the study buddy) every week or month. If you (I) had any problems, he could reply. He actually solved some (problems) in terms of my hardware… From this perspective, I feel someone in the university still cares about you (me), you know? (Participant 3)

#### Constraining

A third theme related to perceptions of stressors (RQ1) was termed constraining. The term captures student perceptions that they may not benefit from their learning to the extent desired. Such negative perceptions were frequently mentioned by participants who had research projects. The perception of constraining was related to the issue of accessibility, which was attributed to difficulties with expectations, prior knowledge, and cultural differences. Although participants may have described their supervisors as being responsible and having a caring attitude, they could also perceive their learning as being obstructed by different cultural backgrounds between themselves and their supervisors:

There are lots of gaps during communication… Just like we Chinese may often say a sentence (with a meaning) from the surface, but my subtext tells you (listeners) another thing (which implies the true intention of the speaker). That is, for a sentence told by they (my supervisor), I have no idea what the subtext the sentence has or if it is just about the surface meaning. (Participant 3)

Participants who were used to the Chinese educational system felt constrained by a lack of guidance particularly when supervisors gave them autonomy in managing their own research:

When a student comes to the university (in China), we will clearly tell them (the student) what our training goals are for you (the student), what classes you (the student) need to take and what goals you (the student) need to achieve. (I) did not have any of these overseas. Then I was asked to select topics and find research questions by myself… I think this is not quite normal. (Participant 4)

#### Stretching

The final theme related to perceptions of stressors (RQ1) was summarized by the theme named stretching. This positive perception was triggered by experiences that were perceived as challenging but achievable. When learning progressed rapidly and developed relevant skills or knowledge, participants reported positive perceptions of stressors even though or even because learning was difficult:

The course which is stressful and rewarding is the most tiring one. For a whole semester, (I) did dozens of – almost 20 quizzes… I think the speed of the course was faster than that of the undergraduate stage (in China). With a single semester, you (students) can learn what you (students) learned as undergraduate students for one or two years. (Participant 10)

I would give that course a high evaluation… Assignments of that course were difficult. We needed to discuss with team members intensively every week —really long discussions. We also needed to design the price, keep an eye on the competitors, minimize the stock, and update the business strategy (in running a simulated company). (Participant 11)

### RQ2. What stress responses are reported?

Four themes were identified as relating to stress responses. These were summarized by the following terms: Withdrawal, Involvement, Surface learning, and Exploration. Two of these themes are eustress (Involvement and Exploration) while the other two are distress (Withdrawal and Surface learning).

#### Withdrawal

A withdrawal was signified by participants reducing their attentional and emotional involvement in their courses and relationships. On occasions this extended to participants thinking about leaving their university:

(When I found I could not hear the sound or see the slides), I would open a game app and find others (many classmates) were (also) logged in. (Participant 13)

It was in a very lonely state at the beginning… It’s so scary… I thought about it (not studying at this university). I wanted to change to another university. (Participant 4)

#### Involvement

Enhanced involvement generally reflected positive perceptions and was characterized by enhanced engagement and increased effort. Participants displayed higher interest in taking classes and expressing themselves and/or being more devoted to their studies:

I felt the teacher was paying attention to us so I was willing to have more communication with him… I would try my best to finish the assignments so that he could be happy. (Participant 12)

I felt my supervisor was kind-hearted. This (the supervisor’s support) would encourage me to finish my research with more effort. (Participant 14)

#### Surface learning

A third theme identified in response to stress was surface learning (Marton and Säljö, 1976). Associated with negative stress perceptions, participants who lacked shared understanding with their supervisors reported the response of surface learning. Despite spending a long time in reading to comply with supervisors’ requirements, they felt confused about their learning and could only have few thoughts and findings:

To discuss with they (the supervisor) every week, you (I) must hand in something (writings). But I did not have a clear goal. I did read books, but I did not know what I was supposed to write… I might start (reading) since Monday, but I still couldn’t write out (my findings on Thursday). (Participant 3)

#### Exploration

The theme of exploration captures a positive response to stress indicated by learning that goes beyond the materials set by teaching staff. Participants’ explorations generally focused on intellectual self-improvement or on cultural differences. This response theme contrasts with that of surface learning which was characterized by a narrow, superficial focus:

(When doing those assignments), I began to think about how I should acquire a (real) business mindset. Maybe (when I work), I could view my tasks in a more macro way. Thinking of products, marketing, and the big commercial environment… instead of focusing on a narrow field. (Participant 11)

When participants engaged with other students to finish demanding tasks, in addition to exploring ways of self-improvement, participants explored cultural diversity and tried to gain a deeper understanding of foreign cultures:

For example, one (NZ) classmate discussed sustainability from (the perspective) of coffee and coffee beans. I thought it was awesome… (Before, I) neglected the efforts of individuals (in contributing to sustainability)… I think it is actually a process of both sides (different classmates) exchanging information and experiencing different cultures. I think it is good. (Participant 1)

(After such group work), you (I) would find it (the problems of team conflicts) might not (be caused by) interpersonal (conflicts); (it is about) culture. That is, it’s not that they (the foreign members) don’t want to understand. In their brains, this knowledge system – this value has been shaped… They are not deliberate. There are indeed cultural differences (or) cognitive differences between us. (Participant 7)

### RQ3. How is the management of distress and eustress reported?

Three overarching strategies were identified for minimizing or managing negative stress and one strategy was identified for managing positive feelings of stress. We named the themes Voice, Emotional support, Seeking alternative learning opportunities, and Goal updating. They are described in turn below.

#### Voice

Students reported voicing their stressful experiences usually by writing to their teachers in the hope that teachers would address the cause of their concerns. This was a strategy associated with some success for distress alleviation:

(Though the teachers’ attitudes could be arrogant), I still sent him emails (to make suggestions)… He accepted (some of my suggestions) and had some changes. I think it is necessary to express my real thoughts and dissatisfaction. (Participant 12)

Connecting with class representatives and seeking official assistance was also reported as an effective approach to making OISs’ voices heard:

They (my classmates) would tell me (that they felt sad because of the teacher’s behaviors), and they would write (their thoughts) in a word document… (I) had these meetings together with the Dean, and (I) would tell them (the Dean) directly. These meetings were effective. (Participant 9)

#### Emotional support

Participants reported that they sought and received emotional support from family members and friends with similar negative experiences. They suggested that such a strategy brought about relief, but the relief was temporary:

They (my parents) will probably reassure you (me) and say “you don’t (need to) worry; you just do it first”… Just after finding a person to tell, (I feel), there is someone who can listen to you (me). (Participant 1)

Every time when (the teacher) did not send the reading materials in time, I would “tucao” (idiom: complain about) that teacher with my (offshore) classmates. We gritted our teeth and managed to carry on. (Participant 12)

#### Seeking alternative learning opportunities

In response to negative stressful experiences, participants reported reducing reliance on the sources of stress and seeking alternative sources or methods of learning. They turned to peers, tutors, or third-party institutions for opportunities to learn:

I actually have fewer contacts with the teacher. Basically, I (study) together with my classmates if I have any questions. Mainly look at what others do and optimize my own methods… A large proportion of the tuition fees’ (value) comes from mutual enrichment between classmates… (My classmates) help to offset (some disappointments because of) the teaching quality. (Participant 7)

I learnt statistics by myself for the doctoral study… I purchased (third-party Chinese) online courses and studied by myself at home. Spent quite a lot of money. (Participant 14)

#### Goal updating

A final stress management strategy identified was goal updating. Participants reported reflecting on their performance and resetting their achievement goals. This strategy was associated almost exclusively with positive stress and related to elevating goals to maintain pressure. There were no substantial instances of participants reducing their goals in response to negative stress. It appeared that participants set a higher goal, such as more effective information searching based on their own achievements, to continually motivate themselves:

Use (higher) efficiency to maintain the positive state… For example, I spent an hour in the task of searching for information today… Then for the second day, I may want to finish it (information searching) within 45 or 30 min. To update my plans and goals every day. (Participant 17)

### RQ4. Are there associations between perceptions of stressors (RQ1), stress responses (RQ2), and stress management strategies (RQ3)?

**Figure 1:**
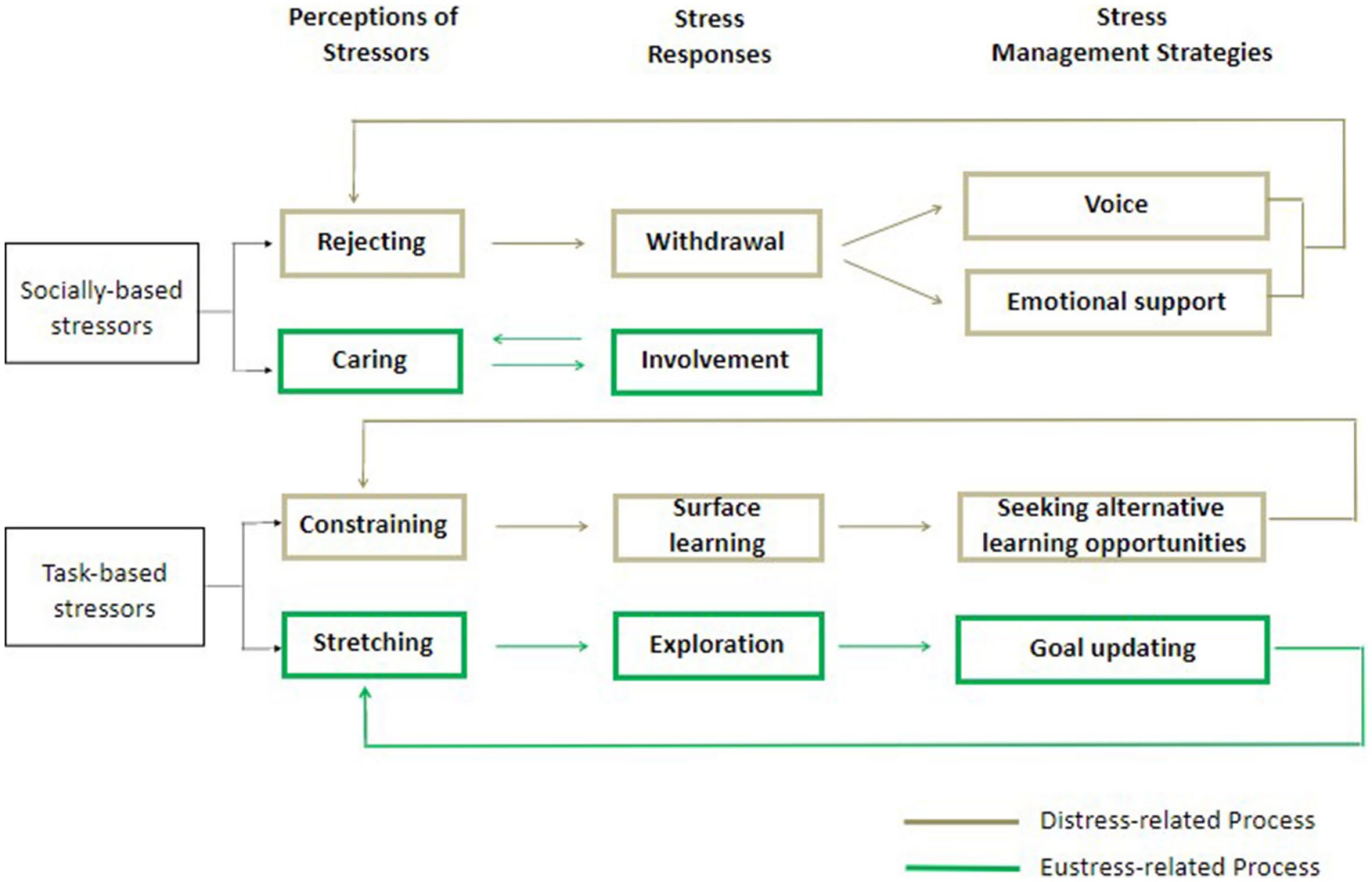
An evidence-based conceptual model.

Participants’ narratives included multiple accounts of causal links between perceptions of stressors, responses, and with management strategies as might be expected from our theoretical framework. In many instances, it was difficult for the authors to identify quotes that related to one research question that did not also include an account relating to the other research questions. When sharing their experiences, participants tended to recall their perception of stressors first and naturally explain their consequential responses and management strategies. It seemed that different stressors evoked perceptions, which correspondingly resulted in dependent stress responses and stress management strategies. The four themes identified in answer to RQ1 were each associated with a unique pattern of responses (RQ2) and management strategies (RQ3). The associations observed are shown in Figure 1 and depict positive and negative experiences originating from socially-based and task-based stressors. Each of the four pathways is described briefly below.

With regards to socially-based stressors, negative perceptions of being rejected were closely associated in participants’ narratives with withdrawal responses. When participants perceived themselves to be rejected during study, they reported their intentions or actions of withdrawing engagement from learning activities. To mitigate the feelings of rejection and the need to withdraw, participants utilized their voice and sought emotional support:

If I found I could not hear the voices (of teachers) or see the slides… there would be less engagement… but we (me and our classmates) would let teachers know there were some issues with the equipment and they (teachers) would adjust in time. (Participant 13)

We were easily distracted (when on-campus people were talking and their voices were too quiet). The effects (of hybrid courses) were not good, personally… Feeling better when there was a friend to have classes together. We could talk with each other when there was dissatisfaction. (Participant 11)

With stressors perceived as positive and as conveying an attitude of caring, participants reported that they responded by actively connecting with teachers and other students. Participants expressed their gratitude and increasing determination to study harder in order not to fail the efforts paid by their teachers. We did not identify any management strategies that sought to prolong eustress resulting from caring perceptions. There seemed to be a virtuous cycle between the caring stressors and the eustress of involvement. When individuals responded to caring stressors with behaviors characterized by involvement, they tended to look forward to having more communication with the caring stressors so that such a positive state could be naturally maintained:

You (I) would be embarrassed (if I) did not take classes seriously. You know, the teacher had already taken care of you (me) so much… (When) she really bought it (a camera) for you (me) to have a clearer view during classes, I was about to cry. I was so touched. (Participant 6)

With regard to negative task-based stressors, when participants talked about their learning process being constrained by vague instructions from teachers, they tended to describe a response of surface learning and sought alternative learning opportunities such as learning from classmates or tutors:

When I felt the teacher did not play a big role… not giving us (students) clear feedback and I had to self-learn, I could be lazy (and think less)… Then I became really confused when doing assignments… Later I would think of solutions because I wanted to pass the course. When I had any questions, I would ask classmates or the tutor. (Participant 1)

Concerning the positive perceptions of being intellectually stretched, participants reported enhanced efforts characterized by exploration and sought to maintain these feelings by engaging in goal updating:

(That assignment) asked you (students) to directly analyze listed companies from the perspective of an analyst… looking for problems and having individual understanding and analysis… I felt the assignment was practical and thought I could use them in my work… (Then) I would put pressure on myself and… give myself new demands. (Participant 18)

A closer examination of these relationships revealed that rather than a uni-directional flow of cause and effect, the flow of influence could be bi-directional. Regarding eustress, for example, there could be a virtuous cycle between caring stressors and the eustress of involvement. Concerning distress, for instance, the usage of the support-seeking strategy (e.g., seeking emotional support from parents) could only partially mitigate students’ distress. Leaving the source (e.g., rejecting stressors) unresolved, students were still likely to experience distress when encountering similar stressors next time.

## Discussion and conclusion

This research set out to address research questions relating to the perception of stressors, specific responses, and management strategies pertaining to OISs’ distress and eustress. We further sought to examine the associations between perception, responses, and management strategies.

This research makes five contributions to the stress literature. It provides empirical evidence for the separate constructs of distress and eustress. It also extends existing stress models (i.e., the Challenge-Hindrance Framework and the Holistic Stress Model) to an educational context and offers new explanations for stress management strategies. Moreover, it demonstrates an innovative usage of the qualitative research method in developing causal models. Finally, it provides insights into the experience of OISs. Each is discussed below.

This research provides empirical evidence for the constructs of distress and eustress, giving weight to the notion that distress and eustress are separate constructs with distinct sources and consequences. By identifying multiple external stressors related to teaching staff, technology, and course design, our research sheds light on the sources of distress or eustress. Therefore, we contribute to the argument that eustress is not simply the absence of distress, and eustress does not always occur whenever individuals do not experience distress (Simmons, 2000; Simmons and Nelson, 2007).

The research both supports and extends the CHF and the HSM in an educational context. Identified tasked-based perceptions of stressors (Constraining and Stretching) echo the proposed hindrance and challenge stressors in the CHF (Lepine et al., 2004). Socially-based perceptions of stressors (Rejecting and Caring) complement the CHF by suggesting that in addition to stress stemming from academic tasks, students may also experience stress relating to interpersonal communication when they recognize themselves as different (e.g., offshore students) from the majority (e.g., on-campus students). Additionally, identified distress (Withdrawal) and eustress (Involvement) in this research corroborate the components of alienation and engagement incorporated by the HSM (Simmons and Nelson, 2007). Responses similar to the distress of Surface learning and the eustress of Exploration, though being studied in the tertiary education context (Johnson and Johnson, 2008; Walker, 2012), have not been thoroughly examined in relation to student stress (Van Slyke et al., 2022). The responses of Surface learning and Exploration thus enhance the HSM and provide future research measuring student stress with more options.

For distress management, we suggest that strategies utilized by participants varied with stress perception and could focus on solving the problem that caused the stress (e.g., Voice) or on alleviating the symptoms or responses to stress (e.g., Emotional support). Based on participants’ narration, strategies addressing the cause were more impactful in terms of minimizing distress than strategies focusing on the symptoms of stress. Though problem-solving strategies have been extensively studied (Lazarus and Folkman, 1984), the scope of the “problem” remains vague; few studies clarify whether the “problem” researched refers to the cause of distress or the troubling symptoms (e.g., Baker and Berenbaum, 2007; Schoenmakers et al., 2015). Thus, differentiated effectiveness between strategies solving causes and symptoms is under-researched and warrants further investigation.

For eustress management, we identified one strategy (Goal updating) for sustaining task-based eustress (Exploration) but did not identify strategies for prolonging socially-based eustress (Involvement). Findings related to eustress management suggest that task-based eustress such as Exploration may need careful management, in that it may subside when individuals overcome difficulties and do not set new goals to continually motivate themselves. Yet for socially-based eustress such as Involvement, no further management strategy may be needed and the eustress itself may be the predictor of future positive perceptions. This is because there seems to be a virtuous cycle between the eustress and associated stressors. Individuals may wish to have more interactions with relevant stressors, and thus such a positive state can be naturally maintained.

This research utilized a qualitative methodology to explore plausible causal relationships related to distress and eustress. Perception-response-management associations identified by this research resonate with the calling for the innovative usage of interpretive qualitative methodology (Lukka, 2014).

Finally, the research provides insight into the stress-related experiences of OISs, a neglected category of students. By making their voices heard, this research identifies a range of educational stressors that have been inadequately examined (e.g., the hybrid teaching format), contributing to researchers’ understanding of student stress in a virtual context (Choo, 2021; Van Slyke et al., 2022).

### Future research directions

Our findings indicate three valuable directions for future research. Firstly, our evidence-based conceptual model (Figure 1) suggests that future research on stress should capture the multi-directional flow of influence between perceptions, responses, and strategies. The dominance of quantitative approaches in this field may have led to the neglect of synergistic and multi-directional influences revealed here through our qualitative approach. Secondly, in addition to external conditions, individual differences could also be considered when investigating stressor perceptions. It is likely that students vary in their views on certain topics, being affected not only by context but also by the interaction between context and personal characteristics (Cabras and Mondo, 2018) and their study habits (Bernardi, 2003). Finally, the topic of eustress remains under-researched, despite, as is suggested here, eustress having constructive behavioral and attitudinal consequences that extend well beyond the pleasure associated with positive responses.

### Practical implications

The research has implications for the preparation of students studying as OISs and implications for institutions that provide such educational opportunities.

Participants’ perceptions of caring or rejecting stressors alert us to the vulnerability and sensitivities of OISs, the crucial role that staff-student relationships have, and the need for teachers to be attuned to students’ emotional well-being. It is highly unlikely that teachers’ behaviors or technological issues occurred through intentional neglect. Teaching students in class and online simultaneously places demands on teachers and systems that institutions may be unprepared for (Choo, 2021). Many participants had experiences of learning in hybrid classes and many of the stressors they identified originated in these classes. Our findings attest to the need to prepare students, teachers, and technologies prior to commencing courses with OISs (Lai et al., 2020).

Students’ perceptions of task-based stressors underline the importance, relevance, and accessibility of teaching and supervision. We suggest that academic staff could profitably attune their attention to students’ feelings about learning and culturally formed expectations as well as attend to students’ comprehension (or lack of it). When students are found to lack academic skills or have expectations inconsistent with educators’ expectations, staff may need to recognized and accommodate the emotional toll as well as provide time for articulating assumptions and helping students reflect on ideas and develop essential skills (Ezebilo, 2012).

Finally, this research has implications for stress management. It may be that students can be helped to develop differentiated adaptive strategies in response to particular stress perceptions. Students can try strategies identified by this research to manage distress or eustress originating from different socially- or task-based stressors.

### Limitations

Study limitations derive from the selection of participants and the data we analyzed. This research was conducted during the period of the COVID-19 pandemic when participants could not go overseas due to visa restrictions. In the main, this was not a situation that either students or their host institutions had anticipated (Wilczewski et al., 2021). This may have influenced their perceptions. Additionally, participants were all Chinese and were postgraduate students; their expectations of study may vary from other students. With regard to data collection and analysis, interviews were used as the only method to collect data, and the researcher did not have opportunities to observe participants or examine objective behavioral data. Additionally, the collected data was only translated by one researcher although as a check, participants had the opportunity to examine Chinese and English transcripts. However, the translation was carried out meticulously and with regard to recent research on transcription and translation techniques. Additionally, the two phases of data collection and analysis served to indicate that the findings are robust.

## Conclusion

This research set out to answer four research questions regarding the perception of stressors, specific responses, stress management strategies, and their associations relevant to the distress and eustress of OISs. Data obtained from semi-structured, in-depth interviews with 18 Chinese participants led to the identification of 12 themes and four causally related pathways connecting perceptions, responses, and management strategies. The research provides empirical support that eustress and distress are separate constructs with distinctive sources, extending existing stress models to an educational context. Additionally, it discloses new insights into students’ stress management and proposes a tentative causal model to advance the understanding of OISs. Findings of this research highlight the impact of context on stress perception, suggesting the importance of analyzing the origins, nature, and management of both eustress and distress in future research.

## Data availability statement

The de-identified data supporting the conclusions of this article will be made available by the authors, without undue reservation.

## Ethics statement

The studies involving human participants were reviewed and approved by Human Participants Ethics Committee of the University of Auckland. The patients/participants provided their written informed consent to participate in this study.

## Author contributions

WG conducted the interviews, data translation, data transcription, data interpretation, and draft writing and editing. SG conducted the data interpretation and draft writing and editing. All authors contributed to the article and approved the submitted version.

## Conflict of interest

The authors declare that the research was conducted in the absence of any commercial or financial relationships that could be construed as a potential conflict of interest.

## Publisher’s note

All claims expressed in this article are solely those of the authors and do not necessarily represent those of their affiliated organizations, or those of the publisher, the editors and the reviewers. Any product that may be evaluated in this article, or claim that may be made by its manufacturer, is not guaranteed or endorsed by the publisher.

## Footnotes

^1^ https://www.iflyrec.com

## References

[ref1] Arbaugh J. B. Godfrey M. R. Johnson M. Pollack B. L. Niendorf B. Wresch W. (2009). Research in online and blended learning in the business disciplines: key findings and possible future directions. Internet High. Educ. 12, 71–87. doi: 10.1016/j.iheduc.2009.06.006

[ref2] Baker J. P. Berenbaum H. (2007). Emotional approach and problem-focused coping: a comparison of potentially adaptive strategies. Cognit. Emot. 21, 95–118. doi: 10.1080/02699930600562276

[ref3] Belk R. W. Fischer E. Kozinets R. V. (2012). “Depth interviews” in Qualitative consumer and marketing research. eds. Belk R. W. Fischer E. Kozinets R. V. (Thousand Oaks, CA: SAGE Publications), 31–56.

[ref4] Bernardi R. A. (2003). Students’ performance in accounting: differential effect of field dependence-independence as a learning style. Psychol. Rep. 93, 135–142. doi: 10.2466/pr0.2003.93.1.135, PMID: 14563040

[ref5] Braun V. Clarke V. (2006). Using thematic analysis in psychology. Qual. Res. Psychol. 3, 77–101. doi: 10.1191/1478088706qp063oa

[ref6] Burke L. A. Miller M. K. (2001). Phone interviewing as a means of data collection: lessons learned and practical recommendations. Forum Qual. Soc. Res. 2, 1–8. doi: 10.17169/fqs-2.2.959

[ref7] Cabras C. Mondo M. (2018). Coping strategies, optimism, and life satisfaction among first-year university students in Italy: gender and age differences. High. Educ. 75, 643–654. doi: 10.1007/s10734-017-0161-x

[ref8] Charles H. Stewart M. A. (1991). Academic advising of international students. J. Multicult. Couns. Dev. 19, 173–181. doi: 10.1002/j.2161-1912.1991.tb00554.x

[ref9] Choi J. Kushner K. E. Mill J. Lai D. W. (2012). Understanding the language, the culture, and the experience: translation in cross-cultural research. Int J Qual Methods 11, 652–665. doi: 10.1177/160940691201100508

[ref10] Choo L. W. (2021). Reflection on supporting offshore international students during the pandemic. New Zealand J. Teachers’ Work 18, 63–68. doi: 10.24135/teacherswork.v18i2.333

[ref11] Crotty M. (1998) The foundations of social research: Meaning and perspective in the research process. 1st. Thousand Oaks, CA: SAGE Publications

[ref12] Ezebilo E. E. (2012). Challenges in postgraduate studies: assessments by doctoral students in a Swedish university. High. Educ. Stud. 2, 49–57. doi: 10.5539/hes.v2n4p49

[ref13] Garbóczy S. Szemán-Nagy A. Ahmad M. S. Harsányi S. Ocsenás D. Rekenyi V.. (2021). Health anxiety, perceived stress, and coping styles in the shadow of the COVID-19. BMC Psychol. 9, 53–13. doi: 10.1186/s40359-021-00560-3, PMID: 33823945 PMC8022303

[ref14] Gibbons C. Dempster M. Moutray M. (2008). Stress and eustress in nursing students. J. Adv. Nurs. 61, 282–290. doi: 10.1111/j.1365-2648.2007.04497.x, PMID: 18197862

[ref15] Guest G. Bunce A. Johnson L. (2006). How many interviews are enough? An experiment with data saturation and variability. Field Methods 18, 59–82. doi: 10.1177/1525822X05279903

[ref16] Halcomb E. J. Davidson P. M. (2006). Is verbatim transcription of interview data always necessary? Appl. Nurs. Res. 19, 38–42. doi: 10.1016/j.apnr.2005.06.001 16455440

[ref17] Hargrove M. B. Nelson D. L. Cooper C. L. (2013). Generating eustress by challenging employees: helping people savor their work. Organ. Dyn. 42, 61–69. doi: 10.1016/j.orgdyn.2012.12.008

[ref18] Holmes A. G. D. (2020). Researcher positionality – a consideration of its influence and place in qualitative research – a new researcher guide. Shanlax Int. J. Educ. 8, 1–9. doi: 10.34293/education.v8i4.3232

[ref19] Hycner R. H. (1985). Some guidelines for the phenomenological analysis of interview data. Hum. Stud. 8, 279–303. doi: 10.1007/BF00142995

[ref20] Johnson R. T. Johnson D. W. (2008). Active learning: cooperation in the classroom. Annu. Report Educ. Psychol. Japan 47, 29–30. doi: 10.5926/arepj1962.47.0_29

[ref21] Lai A. Y. K. Lee L. Wang M. P. Feng Y. Lai T. T. K. Ho L. M.. (2020). Mental health impacts of the COVID-19 pandemic on international university students, related stressors, and coping strategies. Front. Psych. 11, 1–13. doi: 10.3389/fpsyt.2020.584240, PMID: 33329126 PMC7719620

[ref22] Laufer M. Gorup M. (2019). The invisible others: stories of international doctoral student dropout. High. Educ. 78, 165–181. doi: 10.1007/s10734-018-0337-z

[ref23] Lazarus R. S. Folkman S. (1984) Stress, appraisal, and coping. New York: Springer Publishing Company.

[ref24] Le Fevre M. Matheny J. Kolt G. S. (2003). Eustress, distress, and interpretation in occupational stress. J. Manag. Psychol. 18, 726–744. doi: 10.1108/02683940310502412

[ref25] Leech B. L. (2002). Asking questions: techniques for semi-structured interviews. Polit. Sci. Polit. 35, 665–668. doi: 10.1017/S1049096502001129

[ref26] LePine J. A. LePine M. A. Jackson C. L. (2004). Challenge and hindrance stress: relationships with exhaustion, motivation to learn, and learning performance. J. Appl. Psychol. 89, 883–891. doi: 10.1037/0021-9010.89.5.883, PMID: 15506867

[ref27] Liao K. Y. H. Wei M. (2014). Academic stress and positive affect: Asian value and self-worth contingency as moderators among Chinese international students. Cult. Divers. Ethn. Minor. Psychol. 20, 107–115. doi: 10.1037/a0034071, PMID: 24491130

[ref28] Lo Iacono V. Symonds P. Brown D. H. K. (2016). Skype as a tool for qualitative research interviews. Sociol. Res. Online 21, 103–117. doi: 10.5153/sro.3952

[ref29] Lukka K. (2014). Exploring the possibilities for causal explanation in interpretive research. Acc. Organ. Soc. 39, 559–566. doi: 10.1016/j.aos.2014.06.002

[ref30] Marton F. Säljö R. (1976). On qualitative differences in learning: I—outcome and process. Br. J. Educ. Psychol. 46, 4–11. doi: 10.1111/j.2044-8279.1976.tb02980.x

[ref31] Morris M. W. Leung K. Ames D. Lickel B. (1999). Views from inside and outside: integrating emic and etic insights about culture and justice judgment. Acad. Manag. Rev. 24, 781–796. doi: 10.5465/amr.1999.2553253

[ref32] O’Byrne L. Gavin B. Adamis D. Lim Y. X. McNicholas F. (2021). Levels of stress in medical students due to COVID-19. J. Med. Ethics 47, 383–388. doi: 10.1136/medethics-2020-107155, PMID: 33658333

[ref33] Oliffe J. L. Kelly M. T. Gonzalez Montaner G. Yu Ko W. F. (2021). Zoom interviews: benefits and concessions. Int. J. Qual. Methods 20, 160940692110535–160940692110538. doi: 10.1177/16094069211053522

[ref34] Oliver D. G. Serovich J. M. Mason T. L. (2005). Constraints and opportunities with interview transcription: towards reflection in qualitative research. Soc. Forces 84, 1273–1289. doi: 10.1353/sof.2006.0023, PMID: 16534533 PMC1400594

[ref35] Podsakoff N. P. LePine J. A. LePine M. A. (2007). Differential challenge stressor-hindrance stressor relationships with job attitudes, turnover intentions, turnover, and withdrawal behavior: a meta-analysis. J. Appl. Psychol. 92, 438–454. doi: 10.1037/0021-9010.92.2.438, PMID: 17371090

[ref36] Prasetyanto D. Rizki M. Sunitiyoso Y. (2022). Online learning participation intention after COVID-19 pandemic in Indonesia: do students still make trips for online class? Sustainability 14:1982. doi: 10.3390/su14041982

[ref50] Quinlan C. Babin B. Carr J. Griffin M. Zikmund W. G. (2015) Business research methods. 1st. Boston, MA: Cengage Learning EMEA.

[ref37] Schoenmakers E. C. van Tilburg T. G. Fokkema T. (2015). Problem-focused and emotion-focused coping options and loneliness: how are they related? Eur. J. Ageing 12, 153–161. doi: 10.1007/s10433-015-0336-1, PMID: 28804352 PMC5549139

[ref38] Selye H. (1956) The stress of life. New York: McGraw-Hill.

[ref39] Simmons B. L. (2000). Eustress at work: Accentuating the positive. PhD dissertation. Oklahoma State University, United States. Available at: https://shareok.org/bitstream/handle/11244/336405/Thesis-2000D-S592e.pdf?sequence=1

[ref40] Simmons B. L. Nelson D. L. (2007). “Eustress at work: extending the holistic stress model” in Positive organizational behavior. eds. Cooper C. Nelson D. (Thousand Oaks, CA: SAGE Publications), 40–53.

[ref41] Smith R. A. Khawaja N. G. (2011). A review of the acculturation experiences of international students. Int. J. Intercult. Relat. 35, 699–713. doi: 10.1016/j.ijintrel.2011.08.004

[ref42] Tuval-Mashiach R. (2021). Is replication relevant for qualitative research? Qual. Psychol. 8, 365–377. doi: 10.1037/qup0000217

[ref43] Twining P. Heller R. S. Nussbaum M. Tsai C. C. (2017). Some guidance on conducting and reporting qualitative studies. Comput. Educ. 106, A1–A9. doi: 10.1016/j.compedu.2016.12.002

[ref44] Van Slyke C. Clary G. Tazkarji M. (2022). Distress, eustress, and continuance intentions for distance learners. J. Comput. Inform. Syst. Adv. Online Publication 63, 149–161. doi: 10.1080/08874417.2022.2037477

[ref45] Walker D. M. (2012). Classroom assessment techniques: an assessment and student evaluation method. Creat. Educ. 03, 903–907. doi: 10.4236/ce.2012.326136

[ref46] Welch C. Piekkari R. (2006). Crossing language boundaries: qualitative interviewing in international business. Manag. Int. Rev. 46, 417–437. doi: 10.1007/s11575-006-0099-1

[ref47] Wilczewski M. Gorbaniuk O. Giuri P. (2021). The psychological and academic effects of studying from the home and host country during the COVID-19 pandemic. Front. Psychol. 12:644096. doi: 10.3389/fpsyg.2021.644096, PMID: 33897547 PMC8062758

[ref48] Yan K. Berliner D. C. (2013). Chinese international students’ personal and sociocultural stressors in the United States. J. Coll. Stud. Dev. 54, 62–84. doi: 10.1353/csd.2013.0010

[ref49] Zhang Z. Brunton M. (2007). Differences in living and learning: Chinese international students in New Zealand. J. Stud. Int. Educ. 11, 124–140. doi: 10.1177/1028315306289834

